# Nanoclays for Lipase Immobilization: Biocatalyst Characterization and Activity in Polyester Synthesis

**DOI:** 10.3390/polym8120416

**Published:** 2016-12-01

**Authors:** Hale Öztürk, Eric Pollet, Vincent Phalip, Yüksel Güvenilir, Luc Avérous

**Affiliations:** 1 BioTeam/ICPEES-ECPM, UMR 7515, Université de Strasbourg, 25 rue Becquerel, 67087 Strasbourg, Cedex 2, France; medihaleozturk@gmail.com (H.Ö.); phalip@unistra.fr (V.P.); 2 Department of Chemical Engineering, Istanbul Technical University, Maslak, 34469 Istanbul, Turkey; avcibasi@itu.edu.tr

**Keywords:** *Candida antarctica* lipase B, immobilization, sepiolite, montmorillonite, organoclay, ε-caprolactone polymerization

## Abstract

The immobilization of *Candida antarctica* lipase B (CALB) was performed by physical adsorption on both neat and organo-modified forms of sepiolite and montmorillonite. The influence of different parameters, e.g., solvent, enzyme loading, cross-linking, and type of clay support, on immobilization efficiency and catalyst hydrolytic activity has been investigated. The highest hydrolytic activities were obtained for CALB immobilized on organo-modified clay minerals, highlighting the beneficial effect of organo-modification. The esterification activity of these CALB/organoclay catalysts was also tested in the ring-opening polymerization of ε-caprolactone. The polymerization kinetics observed for clay-immobilized catalysts confirmed that CALB adsorbed on organo-modified montmorillonite (CALB/MMTMOD) was the highest-performing catalytic system.

## 1. Introduction

Lipases (E.C 3.1.1.3), members of the hydrolase family, are esterases that can hydrolyze triglycerides (or esters) to glycerol and fatty acids at the water-oil interface [1]. Interestingly, in certain cases, lipase-catalyzed hydrolysis in water can be easily reversed in non-aqueous media to ester synthesis or transesterification [2]. This specific behavior paved the way for the development of lipase-catalyzed ring-opening polymerization (ROP) of lactones, cyclic diesters (lactides), and cyclic carbonates to produce aliphatic polyesters or polycarbonates [3,4].

The immobilization of enzymes is often carried out to increase their activity and stability and to allow easier separation from the reaction mixture [5,6]. Immobilization can be performed either by simple physical adsorption on the support or by covalent bonding between the enzyme proteins and the carrier surface. However, covalent anchoring may affect the enzyme activity due to possible difficulties for the enzyme to change its structure to accommodate the specific organic substrate [6]. Thus, in recent years, adsorptive enzyme immobilization techniques have been intensively studied on account of their intrinsic advantages, i.e., simplicity, potential reversibility, and the possibility of high activity retention due to the absence of chemical modification [6]. The physical adsorption and binding of the enzyme depend on the nature of the surface and may be the result of ionic (electrostatic) interactions, hydrophobic affinity, Van der Waals attractive forces, hydrogen bonding, or a combination thereof [7]. Immobilization of lipases is, thus, often a result of adsorption through hydrophobic interactions between the enzyme and the support [8].

Among lipases, *Candida antarctica* lipase B (CALB) is the most commonly used for many industrial applications due to its high enantioselectivity, wide range of substrates, thermal and organic solvent stability [9].

So far, CALB has been immobilized onto various types of supports, for example, porous polymeric particles of polypropylene [10], polystyrene [11] or acrylic resin [12], mesoporous silica [13], diatomite [12], alumina [14], activated carbon [15], chitosan [16], and agarose [16,17].

The most commonly used catalytic system is the commercially available Novozym^®^-435, in which CALB is physically immobilized on a macroporous acrylic resin. The polymeric nature of the support presents certain disadvantages, such as low organic solvent stability, limited thermal stability, and low mechanical resistance.

Recently, the use of phyllosilicates as inorganic solid supports for the immobilization of enzymes has gained increased interest. Nanoclays such as sepiolite [18,19,20], montmorillonite [19,21,22,23,24] and bentonite [20,25,26,27,28,29] have been tested for enzyme immobilization. The use of nanoclays as lipase carriers can provide numerous advantages, in particular, high specific surface areas, facile water dispersion/recuperation, high swelling capacities, and high mechanical resistance [19].

Montmorillonite is a crystalline, layered silicate mineral, displaying intercalation properties and a large surface area (ca. 700 m^2^/g) for the adsorption of organic and inorganic moieties [21]. Sepiolite is a non-swelling, porous clay mineral. Compared to montmorillonite it has a lower, but still significant, specific surface area (ca. 400 m^2^/g). Sepiolite particles have a needle-like morphology and a high density of silanol groups (–SiOH). These functional groups present an interesting potential for the adsorption of lipase. The hydrophilic character of the neat clays can be modified by simple cation exchange, where hydrated inorganic cations present in the native clay structure can be replaced by organic cations to render the clay more hydrophobic. The resulting, so-called, organoclays are more compatible with organic molecules such as polymers or enzymes. It has been reported that changes to the hydrophobicity of the support, e.g., through organo-modification, could alter the strength and amount of lipase adsorption and, therefore, the final catalytic activity of the lipase [28].

In a recent study, we showedthat CALB immobilized on sepiolite and montmorillonite and on their organo-modified counterparts can be efficient catalytic systems for the enzymatic ring-opening polymerization (eROP) of ε-caprolactone and lactides [30,31]. The present work, thus, aimed at studying the influence of clay morphology and organo-modification on CALB adsorption. Immobilization parameters such as enzyme loading, medium, glutaraldehyde treatment, and catalyst recovery procedures were investigated in detail. The resulting CALB/clay catalytic systems were characterized and their performance in terms of hydrolytic activity and ε-caprolactone polymerization efficiency were evaluated and compared to Novozym^®^-435.

## 2. Materials and Methods

### 2.1. Materials

Commercially available neat sepiolite (SEP) was supplied by Tolsa (Madrid, Spain) under the trade name Pangel^®^ S9. Neat montmorillonite (MMT) clay and Cloisite^®^ 30B, the organo-modified montmorillonite (MMTMOD) modified by tallow alkyl methyl *bis*(2-hydroxyethyl) ammonium cations, were supplied by Southern Clay Products (Gonzales, TX, USA). Organo-modified sepiolite (SEPMOD), was prepared from neat sepiolite and tallow alkyl methyl *bis*(2-hydroxyethyl) ammonium (Ethoquad O/12) by the process described below.

The free form of *Candida antarctica* lipase B (CALB) in aqueous solution, was kindly provided by Novozymes (Bagsværd, Denmark) and used after dialysis and lyophilization. Novozym^®^-435 (NOV-435), an acrylic resin-immobilized form of CALB, was purchased from Sigma-Aldrich (Saint-Quentin Fallavier, France). Also ε-caprolactone (99%, Acros, Illkirch, France) was dried and stored over molecular sieves before polymerization. Anhydrous toluene was freshly distilled over sodium under nitrogen atmosphere. Bovine serum albumin (BSA) and *p*-nitrophenyl butyrate (*p*NPB), both from Sigma Aldrich, and dye reagent from Bio-Rad (Marnes-la-Coquette, France) were used for the protein assay and lipase hydrolytic activity measurements.

### 2.2. Methods

#### 2.2.1. Surface Modification of Clay Minerals

To modify the neat sepiolite surface with the same organo-modifier as used for Cloisite^®^ 30B, namely tallow alkyl methyl *bis*(2-hydroxyethyl) ammonium salt, the following procedure was used. An amount of 5 g of sepiolite (Pangel^®^ S9) was suspended in 250 mL of deionized water and dispersed in an ultrasonic bath for 4 h at 60 °C. To this, 0.3 g of Ethoquad O/12 (tallow alkyl methyl *bis*(2-hydroxyethyl) ammonium salt) was added to the sepiolite dispersion and the mixture sonicated for 1 day at 60 °C. The mixture was then filtered and washed with 2 L of distilled water at 60 °C to remove the formed NaCl salt and excess ammonium. Finally, the resulting sludge was lyophilized to recover the organo-modified sepiolite as a dry powder.

#### 2.2.2. Immobilization of Lipase

For the immobilization process, varying quantities of dialyzed and lyophilized CALB enzyme were added to previously weighed clay supports (corresponding to 7.5, 13.5 and 27 mg of enzyme per gram of clay) and dispersed in 0.1 M sodium phosphate buffer solution (pH 7.0). Mixtures were stirred for 2 h at 4 °C and the resulting suspensions were centrifuged at 6000 rpm for 20 min at 4 °C. The centrifugation pellets were washed twice by the buffer solution to remove loosely-bound enzymes. The supernatant was tested for protein content and enzymatic activity. The CALB immobilized inorganic supports were recovered as a paste due to the swelling of clay minerals in the buffer solution. The pastes were lyophilized in a Christ-Alpha 2-4 LD lyophilizer (Martin Christ, Osterode, Germany) for 1–2 days, depending on the sublimation rate of the aqueous buffer. The resulting immobilized enzyme derivatives, obtained as powders, were stored at 4 °C under vacuum in a desiccator prior to their usage as catalysts in polymerization reactions.

The efficiency of immobilization was evaluated according to Equation (1):

(1) $$\mathrm{Immobilization}\;\mathrm{efficiency}\;\left(\%\right)=100\;\times \;\frac{\mathrm{amount}\;\mathrm{of}\;\mathrm{fixed}\;\mathrm{protein}}{\mathrm{amount}\;\mathrm{of}\;\mathrm{loaded}\;\mathrm{protein}}\;,$$

from the ratio between the determined bound protein content (see Section 2.2.4) and the initial protein loading.

The average standard deviation on the calculated mean values of immobilization efficiency was estimated to be ±3%.

#### 2.2.3. Glutaraldehyde Treatment

The various CALB immobilized clay supports were incubated with 0.5% (*v*/*v*) glutaraldehyde solution in 25 mM sodium phosphate buffer at pH 7.0 and 25 °C for 1 h with mild stirring. The glutaraldehyde treatment is expected to result in a modification of the primary amines of the enzyme and the functional groups of the support. Samples were centrifuged at 6000 rpm for 20 min at 4 °C. The centrifuge pellets were washed with 25 mM sodium phosphate buffer (pH 7.0) to remove excess glutaraldehyde (GA) and the process repeated three times. The treated supports were incubated for an additional 24 h at 25 °C to achieve greater cross-linking density between the enzyme and the support.

#### 2.2.4. Determination of Protein Content

To determine the efficiency of lipase immobilization on the clay supports, protein content was analyzed at each step. Protein content was estimated according to Bradford’s method using bovine serum albumin (BSA) as a standard [32]. The amount of bound protein was calculated from the difference between the concentration of loaded protein and that present in the supernatants, determined by Bio-Rad protein microassay procedure (Bio-Rad, Marnes-la-Coquette, France). Absorbance values (OD595) were measured at 595 nm. All readings were carried out in duplicate. The protein concentration for each sample was calculated using Equation (2):

Protein concentration (μg/mL) = (OD595 − 0.0053)/0.0432,(2)

obtained from a plotted BSA standard curve.

#### 2.2.5. Measurement of Lipase Hydrolytic Activity

This assay was performed by measuring the increase in absorbance at 410 nm, produced by the release of *p*-nitrophenol (*p*NP) that results from the hydrolysis of the *p*-nitrophenyl butyrate (*p*NPB) substrate. Initially, 100 μL of enzyme solution was added to a spectrophotometer cuvette containing 900 μL of substrate solution (0.5 mM *p*NPB in 25 mM sodium phosphate buffer; pH 7.0). Absorbance values were recorded at 410 nm with a Genesys 10S Bio UV-Vis spectrophotometer (Thermo-Fisher, Illkirch, France) at regular time intervals. Between each measurement, the cuvette was continuously agitated. Measurements were performed in triplicate. The enzymatic activity was deduced from the slope of the linearized curve of absorbance vs. time and the molar extinction coefficient of *p*NP. One unit of enzyme activity (U) was defined as 1 μmol of *p*NP liberated in 1 min by 1 mg of CALB at 25 °C (1 U/mg = 1 μmol *p*NP/min·mg). The average standard deviation of the calculated mean values of hydrolytic activity was estimated to be ±10%.

#### 2.2.6. Applicability of Immobilized Lipase for Esterification Reactions: ε-CL Polymerization Set-Up

All reactions were carried out in dry toluene at 70 °C. Pre-determined amounts of catalyst, immobilized CALB derivatives (i.e., CALB immobilized on nanoclays) or NOV-435, were introduced into previously dried Schlenk tubes under dry nitrogen atmosphere. The tube was immediately capped with a rubber septum. 10 mL of toluene and 3 mL of ε-CL were transferred with a syringe through rubber septum caps. The reaction tube was then immersed in a heated oil bath and the polymerization reaction was allowed to proceed. Aliquots were withdrawn at specific time intervals to monitor the progress of the polymerization reaction. Reactions were terminated by dissolving the reaction mixture in chloroform and, when necessary, filtering out the catalyst. Chloroform in the filtrate was then removed by rotary evaporation at 35 °C. The polymer in the resulting concentrated solution was precipitated in cold methanol (previously stored at −20 °C). The polymer precipitate was separated by filtration and dried overnight at 30 °C under vacuum.

### 2.3. Characterization Techniques

#### 2.3.1. Nuclear Magnetic Resonance (NMR)

ε-CL polymerization was monitored by proton nuclear magnetic resonance (^1^H NMR) spectroscopy to determine monomer conversion and the number average degree of polymerization. ^1^H NMR spectra were recorded in CDCl_3_ on a Bruker NMR spectrometer (Bruker, Wissembourg, France) at 300 MHz. The percentage conversion of monomer to polymer was determined from the integral ratios of the characteristic monomer and polymer peaks. The average standard deviation of the calculated mean values of monomer conversion was estimated to be ±5%.

Number-average molar masses (*M*_n_) of polycaprolactone (PCL) were calculated from the degree of polymerization (*DP*_n_), which was determined from the integral ratios of the characteristic peaks of the polymer chains and chain-ends (*M*_n_ PCL = *DP*_n_ × 114 g/mol).

#### 2.3.2. Scanning Electron Microscopy (SEM)

The surface morphology of the CALB/clay derivatives and clay minerals were characterized by scanning electron microscopy (SEM). Samples were previously coated with a layer of gold to minimize charging effects. Analyses were performed on a Philips XL30 SEM apparatus (Philips, Eindhoven, The Netherlands) with accelerating voltage of 20 or 25 kV and images were recorded at different magnifications.

#### 2.3.3. X-ray Diffraction Analysis (XRD)

X-ray diffraction (XRD) patterns of samples were recorded on a Siemens/Bruker D-5000 powder diffractometer (Siemens/Bruker, Champs-sur-Marne, France) with Cu Kα radiation source (λ = 0.1546 nm). The incidence angle was varied between 2° and 9° with step size of 0.010° and step time of 4 s (scanning rate 0.15°·min^−1^). The basal spacing of neat and modified clay minerals, before and after enzyme immobilization, was calculated according to Bragg’s law, given in Equation (3):

λ = 2 × *d*_001_ × sinθ, (3)

where λ is the wavelength of the source, *d*_001_, the interplanar distance between 001 planes, and θ, the diffraction angle.

## 3. Results and Discussion

### 3.1. CALB Immobilization: Influence of the Type of Clay Support

In the first batch of immobilization, sepiolite (SEP) and montmorillonite (MMT) nanoclays and their organo-modified forms (SEPMOD and MMTMOD) were used as lipase supports. The resulting CALB/clay catalysts were compared in terms of adsorbed enzyme content and hydrolytic activity.

For these four types of clay minerals, various lipase loadings were tested (respectively 7.5; 13.5 and 27 mg of protein per gram of clay). In the sample coding, the letter L stands for CALB immobilized on the different types of clay, the number 1, 2 or 3 stands for the corresponding amount of enzyme loading and the last letter (a, b) denotes the catalysts resulting from different batches of immobilization. Table 1 presents the percentage of immobilized enzyme and the determined specific hydrolytic activity of the CALB/clay systems for the *p*NPB substrate.

The determination of protein content showed that, with the exception of SEPMOD, excessive enzyme loading results in lower immobilization efficiency, both for sepiolite and montmorillonite clay minerals. It was, thus, determined that the optimal lipase content for immobilization was 7.5 mg of protein per gram of clay mineral. While it is well known that increasing the amount of enzyme loading can lead to higher amounts of immobilized enzyme, the formation of thicker layers of adsorbed lipases often results in a loss of specific activity due to some enzymes becoming “buried” and, thus, being less accessible to the substrate [33]. This was visible here, with all systems displaying a decrease in specific activity as enzyme loading increased (except for SEP, which showed very low activity regardless of the loading).

The results of this immobilization study highlighted the positive impact of organo-modification of the clay surface on lipase immobilization efficiency and more importantly on hydrolytic activity. The results obtained here for organo-modified clay minerals were also in perfect agreement with the findings of Tzialla et al. [25]. This was particularly marked for sepiolite-based systems, which showed great enhancement in their hydrolytic activity, with an increase from ca. 40 to 200 U/mg CALB, when using their organo-modified forms for lipase immobilization (Table 1). It can be assumed that enzyme molecules preferentially interacted with the organo-modifier (ammonium salt) when using organo-modified sepiolite as compared to neat sepiolite, thus, providing better conformational positioning and accessibility to the active center of the enzyme, as also reported by Sedaghat et al. [18]. These differences in lipase immobilization and hydrolytic activities could be due to the differences in the electrostatic interactions between the enzyme, the various clay surfaces, and the functional groups of the organo-modifier. It is also possible that specific surfaces may direct enzyme molecules towards a particular orientation so that the active site of the enzyme becomes more accessible to the substrate, thus, enhancing its activity.

The loss of activity of CALB after its immobilization on neat sepiolite can be explained by a number of factors; for example, the possible strong interactions between the enzyme amino acids, essential for bonding or catalysis, and the surface of natural clay mineral. The steric hindrance to the approach of the substrate, the disruption of the 3D structure of the enzyme protein, and the diffusional limitations for solute transport near particles may also play a role in the observed behavior [18]. Due to immobilization, the active center of the lipase is in close vicinity to the surface of the clay support and this may create a more hydrophobic environment around the active center compared to the aqueous medium surrounding the free enzyme. The hydrophobicity of the support or its internal morphology can thus change the adsorption strength of the lipase and the structure of the active center surroundings and subsequently the final catalytic features of the lipase [34].

Fernandez-Lafuente et al. [17] suggested that the adsorption of lipases may be influenced by some factors more complex than just the hydrophobicity of the support. Finally, they concluded that the amount of lipase adsorbed is higher for more hydrophobic supports. This can explain the higher activity of MMTL catalytic systems over SEPL ones (e.g., 117 U/mg CALB for MMTL1a vs. 37 U/mg CALB for SEPL1a) (Table 1), as CALB is expected to show higher enzymatic activity when adsorbed on more hydrophobic supports, such as montmorillonite, compared to sepiolite, which is more hydrophilic. Another possible explanation regarding the lower activity of the sepiolite-based systems is the difference in terms of available surface area and location of the immobilized enzyme. On account of its higher surface area (ca. 700 vs. ca. 400 m^2^/g for sepiolite) and good swelling capacity, MMT may exhibit greater adsorption capacity with thinner layers of immobilized enzymes, resulting in more active sites available for catalysis.

Moreover, both unmodified clay minerals show a global negative charge on their surface, which may be not fully appropriate for lipase immobilization and further catalytic activity. Indeed, the immobilization procedure was performed in a pH 7.0 buffer solution, which is above the lipase isoelectric point (pI 6.0). Under these conditions, one may assume that the enzyme globally bears a slightly negative charge and thus may show limited interactions (or even some repulsion) with the clay mineral surface. On the other hand, organo-modified supports should present an apparent positive charge on their surface due to the ammonium salts, which could interact favorably with the globally negatively charged enzyme at pH 7.0, thus, promoting immobilization. Furthermore, it can also be supposed that the adsorption mechanism for silicates would be enhanced by interactions between surface silanol groups and the positive charges (protonated lysine) of the enzymes. However, using such a protonated form of the enzyme to obtain more efficient lipase immobilization would require the use of acidic conditions during immobilization, which may alter the structural integrity of the clay mineral.

Thus, even if the precise explanation is not yet fully understood, it is evident that the organo-modification of the clay surface dramatically improves the efficiency of the immobilized lipase catalysts. In addition, catalytic systems based on montmorillonite appear to perform better than those based on sepiolite.

### 3.2. Influence of Glutaraldehyde Treatment

Glutaraldehyde, which is known for its capacity to cross-link proteins, was used in the next step to increase the stability and specific activity of the immobilized catalysts. GA is a linear 5-carbon dialdehyde widely used for enzyme immobilization. However, its structure in aqueous solutions and its corresponding cross-linking mechanism remain open to debate [35]. The cross-linking of proteins to a solid support or between protein molecules (support-free) usually makes use of the ε-amino groups of lysine residues which, in most cases, are not involved in the catalytic activity of the enzyme. This allows moderate cross-linking to preserve the conformation of the protein and its catalytic activity [36]. As well as enhancing thermal stability, immobilization of enzymes on supports by adsorption followed by cross-linking often improves the operational stability of the enzyme [6].

There are two main strategies for cross-linking proteins using glutaraldehyde, i.e., the use of supports previously activated by glutaraldehyde or the treatment of previously adsorbed proteins with glutaraldehyde [37]. The first strategy limits the chemical modification of the enzyme to those functional groups of the protein that are involved in the immobilization, while in the second case the entire protein surface may be modified. However, the first strategy mainly leads to low densities of weak multi-point covalent attachments [17,37]. Thus, the second approach is often preferred as, under mild conditions and upon initial adsorption of the enzyme to the support, all primary amino groups of the enzyme and some functional groups of the support may be activated with one molecule of glutaraldehyde. It has been suggested that in this form, GA bound to the ε-amino groups of the enzyme’s lysine residues could covalently react with another GA molecule bound to the activated support and create a multi-point covalent enzyme-support attachment [37].

As can be seen from the results in Table 1, GA treatment results in an enhancement in hydrolytic activity for catalytic systems with organo-modified supports. This effect was most significant for organo-modified montmorillonite-based catalysts (e.g., from 165 to 283 U/mg CALB for MMTMODL1a). On the contrary, the catalytic activities of neat montmorillonite-based systems remained almost unchanged. The positive effect of the glutaraldehyde treatment was not so obvious for catalytic systems based on organo-modified sepiolite, which only showed a slight increase in hydrolytic activity after cross-linking with GA. Moreover, due to the surface properties of unmodified sepiolite, GA treatment led to the formation of a structure that did not promote the catalytic activity of the immobilized lipase and even induced a negative effect on the hydrolytic activity of the catalyst. This could be due to intense cross-linking of thick layers of adsorbed enzymes, resulting in a lower accessibility of the substrate to the active sites.

In order to explain the activity increase observed for free CALB after GA treatment, it can be hypothesized that a large complex composed of dissolved enzyme molecules linked to each other was formed. As a result, an over-concentrated micro-environment in the substrate solution (*p*NPB) could have been created around this complex, providing a higher diffusion of substrate molecules and, thus, an increased probability of an enzyme-substrate interaction. 

### 3.3. Surface Characterization of CALB/Clay Catalysts by SEM

**Figure 1:**
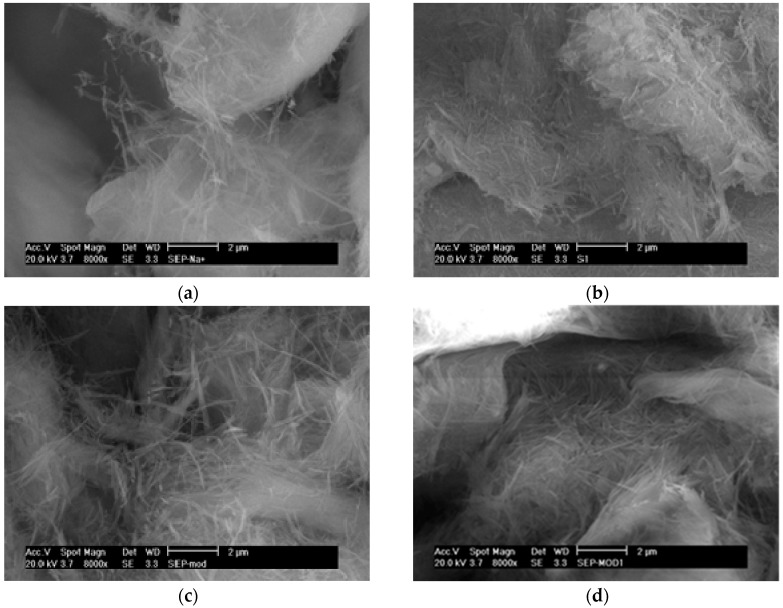
SEM images of (**a**) neat SEP; (**b**) neat SEP after the immobilization of CALB (SEPL); (**c**) SEPMOD; (**d**) SEPMOD after immobilization of CALB (SEPMODL), at 8000× magnification.

**Figure 2:**
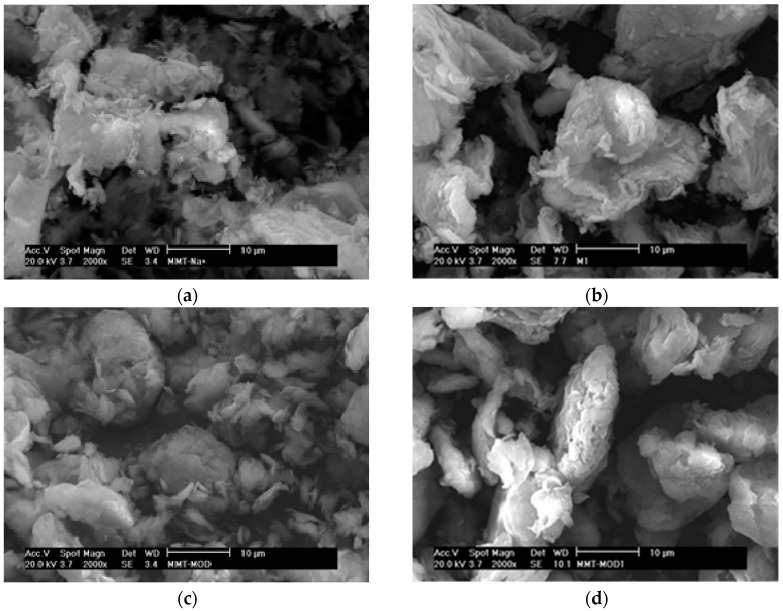
SEM images of (**a**) neat MMT; (**b**) neat MMT after immobilization of CALB (MMTL); (**c**) MMTMOD; (**d**) MMTMOD after immobilization of CALB (MMTMODL) at 2000× magnification.

As the main variations in catalyst activities could be assigned to differences in the structure and surface properties of the clay minerals, morphological studies of the various systems were carried out by scanning electron microscopy (SEM). SEM images of the different clay minerals and CALB/clay catalysts at two different magnifications are shown in Figure 1 and Figure 2.

In the case of neat sepiolite, large aggregates formed by the needle-shaped nanoclay could be observed as well as individualized needles (Figure 1a). Enzyme immobilization on the neat sepiolite maintained the highly aggregated morphology but the individual needles that were observed previously now seem to be embedded in the immobilized enzyme on the clay surface (Figure 1b).

After organo-modification, SEM analysis seems to show that, while some sepiolite aggregates remain, an increased number of individual needles can be observed (Figure 1c). Similarly to SEPL, the SEM image of the organo-modified sepiolite with immobilized lipase seems to show that individual needles are, once again, embedded in the adsorbed enzyme layer at the clay surface (Figure 1d). This was likely due to the non-swelling character of the sepiolite, resulting in the organo-modification and enzyme adsorption to occur only at the surface of the clay mineral. Following cross-linking with GA, the SEM observations did not show significant differences between GA-treated and non-treated systems. Individual sepiolite needles were still embedded in cross-linked enzyme and large clay aggregates were still present (Figure S1).

The morphologies observed for montmorillonite and its derivatives were very different than those of sepiolite. Initial SEM analysis reveals large aggregates of clay platelets stacks for samples of neat MMT (Figure 2a). These clay platelets seem to be more visible after immobilization of CALB on the surface (MMTL). In fact, the aggregates appear to exhibit a smoother surface after lipase immobilization (Figure 2b). The morphologies observed for MMTMOD highlighted the benefit of surface organo-modification, as the aggregates seemed to be smaller (Figure 2c). After enzyme immobilization, the clay platelets could be detected in these aggregates and the adsorption of lipase on the external surface appeared to result in a smoothing of the surface of the clay stacks (Figure 2d). After GA treatment, no significant differences could be observed from the SEM images except at higher magnification where smoother surfaces were visible on the montmorillonite platelets that appeared to be fully embedded in the cross-linked enzyme (Figure S2).

Thus, these SEM observations confirm the expected location of immobilized lipase at the surface of the clay aggregates, as well as reinforcing the benefits of clay organo-modification, which appears to result in smaller aggregates and, thus, higher surface areas for enzyme immobilization.

### 3.4. Characterization of CALB/Clay Catalysts by XRD

**Figure 3:**
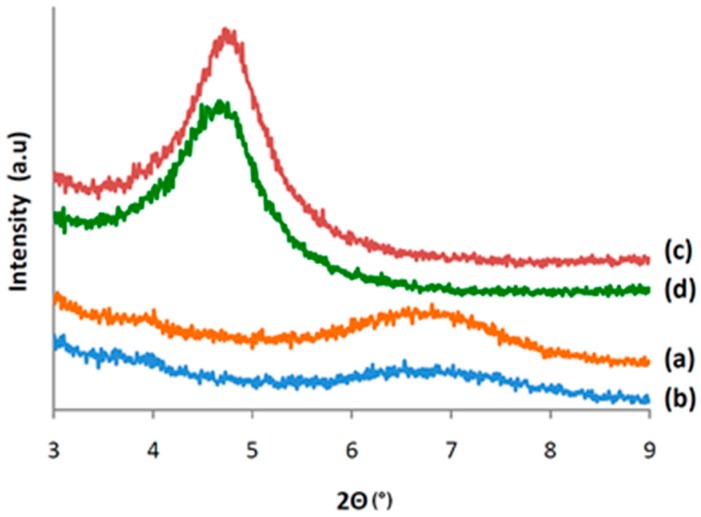
X-ray diffraction (XRD) patterns of (**a**) neat montmorillonite (MMT); (**b**) CALB-immobilized neat montmorillonite (MMTL1); (**c**) organo-modified montmorillonite (MMTMOD); (**d**) CALB-immobilized organo-modified montmorillonite (MMTMODL1).

The CALB immobilized clays were characterized by XRD to determine the location of the adsorbed enzyme (Figure 3). Modified and neat clay minerals were also compared to determine the effect of organo-modification and lipase immobilization on the structure of the clay mineral.

As expected, the characteristic 001 peak of neat montmorillonite was shifted to lower angles due to the expansion of the interlayer space by the intercalation of the alkylammonium molecules of the organo-modifier. The basal spacing of MMT, which is 1.38 nm (2θ = 6.8°), was expanded to 1.88 nm (2θ = 4.8°) after organo-modification.

Moreover, the adsorption of the enzyme onto both neat and organo-modified montmorillonite seemed to be limited to the external surface of the clay, as only a slight decrease in the reflection intensities of MMT and MMTMOD was observed and no reflection shift was detected (Figure 3). Taking into consideration the specific dimensions of CALB (ca. 3 × 4 × 5 nm^3^), the enzyme cannot be intercalated between clay nanosheets. A likely scenario is the intercalation of some amino acid residues while the polypeptide backbone remains outside of the interlayer space [25].

**Figure 4:**
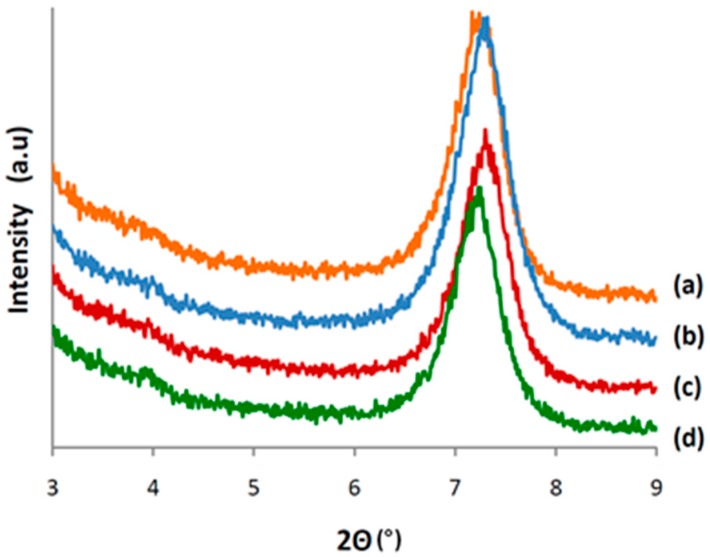
XRD patterns of (**a**) neat sepiolite (SEP); (**b**) CALB-immobilized neat sepiolite (SEPL1); (**c**) organo-modified sepiolite (SEPMOD); (**d**) CALB-immobilized organo-modified sepiolite (SEPMODL1).

In the case of sepiolite, both enzyme immobilization and organo-modifier adsorption appeared to occur on the external surfaces or edges of the clay mineral as no shift in reflection angles was observed (Figure 4). The basal spacing of SEP (1.21 nm; 2θ = 7.3°) remained unchanged confirming the already known fact that the alkylammonium salt cations used for its modification could not penetrate the pore spaces of the sepiolite particles and only occupied the external surface [38]. Moreover, due to the dimensions of CALB, the SEP internal channel is not available to the lipase. Thus, the immobilization can only occur at the external surface of the SEP needles.

### 3.5. CALB Immobilized on Organo-Modified Clays: Catalytic Performance Study

The previous results (Section 3.1) highlighted that CALB/organoclay catalysts prepared with 7.5 mg of protein loading per gram of clay showed the highest immobilization efficiency and hydrolytic activity. Furthermore, cross-linking with glutaraldehyde enhanced the lipase hydrolytic activity for catalytic systems based on organo-modified clays. Thus, a new series of immobilizations was carried out on these organoclay supports, with a loading of 7.5 mg protein per gram of clay mineral, but with higher amount of material (lipase + clay) to obtain adequate quantities of derivatives to be used as catalysts. The aim was to analyze the potential effects of GA and lyophilization on the hydrolytic activity of the lipase and on its performance in the polymerization reaction (Table 2).

#### 3.5.1. Influence of Lyophilization on Hydrolytic Activity

It is important to point out that some variation in terms of specific activities can be observed from one batch of immobilization to another for the same type of support. Besides the typical variations in immobilization procedure, these differences can also be due to dilutions performed for the lipase activity assay and slight differences in ambient temperature during the assay readings. Thus, all lipase hydrolytic activity assays were performed in triplicate. From the results obtained on different batches, the average standard deviation of the calculated mean hydrolytic activity was estimated to be ±10%.

As these CALB/clay systems were to be used as catalysts in polymerization reactions in organic media, a lyophilization step was performed to eliminate the water from the systems prior to use. At the end of the conventional immobilization procedure used thus far, the derived catalysts in the form of a paste were separated into two equal parts. One part was directly lyophilized (e.g., SEPMODL1b) while the other part was treated with GA and then lyophilized (SEPMODL1b G). In this step, a drastic loss of activity occurred which is likely due to a significant loss of enzyme structural water resulting in its denaturation (Table 2). During the freezing step prior to lyophilization, the exposure of proteins to the ice-water interface may have also led to their denaturation [39].

For the non-lyophilized catalysts, a significant increase in hydrolytic activity was detected after GA treatment, especially for the modified montmorillonite-based system (from 174 to 318 U/mg CALB), as already presented and discussed in Section 3.2. However, no significant protective effect on the enzymes from the GA treatment prior to lyophilization could be seen. All derivatives, GA treated or not, lost an important part of their hydrolytic activities after lyophilization.

#### 3.5.2. Esterification Activity Test: Polymerization of ε-Caprolactone

To study the efficiency of the CALB/clay catalysts for esterification reactions, enzymatic ring-opening polymerizations (ROP) of ε-CL were performed by adding 300 mg of the lyophilized immobilized enzymes to the reaction medium. The influence of glutaraldehyde treatment, immobilization conditions, as well as the biocatalyst drying and recovery were investigated.

In the case of glutaraldehyde treatment, for both montmorillonite- and sepiolite-based systems, no significant differences in terms of polymerization kinetics and PCL average molar masses were observed between untreated and GA-treated catalysts. Thus, only the catalytic performances of the GA-treated immobilized systems for ε-CL ROP and their comparison with the NOV-435 commercial catalyst are presented (Table 3).

The results from these experiments have already been presented and discussed in a previous study [30] and will only be briefly summarized below to facilitate further comparison. The reaction kinetics was monitored until monomer conversion reached at least 80% (up to eight days for some systems). However, for the sake of comparison, only the data obtained after 24 h are presented in Table 3. For SEPMODL1b G, after 24 h, the monomer conversion was 15% with a *M*_n_ for PCL of 1700 g/mol. The reaction was quite slow and eight days were needed to reach a monomer conversion of 80% with the average molar mass of PCL remaining rather low (5000 g/mol).

Interestingly, while SEPMODL1b G and MMTMODL1b G showed almost similar hydrolytic activities for the *p*NPB substrate in aqueous solution, their catalytic behavior for ε-CL polymerizations in toluene was completely different. With regard to the reaction kinetics for MMTMODL1b G, one can clearly see that the polymerization proceeded faster (ca. 70% monomer conversion after 24 h) with longer PCL chains, compared to the catalytic system based on organo-modified sepiolite. When comparing NOV-435 to the MMTMODL1b G, the latter catalytic system showed lower polymerization rates and products of lower molar masses were formed. However, a drastic decrease in molar mass of PCL chains was observed with NOV-435 with the addition of MMTMOD to the reaction medium (ca. 9000 g/mol), although conversion remained almost the same. This was due to the presence of hydroxyl groups at the surface of MMTMOD acting as co-initiators during the polymerization and initiating higher numbers of growing chains resulting in PCL with lower molar masses [30,40].

It is worth pointing out that the commercial catalyst NOV-435 contains 10 wt % protein, thus introducing 50 mg of this catalyst corresponds to the addition of 5 mg of protein (i.e., a CALB/ε-CL ratio of 0.17 wt %). By comparison, with the addition of 300 mg of MMTMODL1b G, approximately 2.8 mg of enzyme was introduced into the reaction medium. As can be seen from the CALB-to-CL ratio given in Table 3, the amount of enzyme involved in the reaction was much lower for the clay-immobilized lipase system (CALB/ε-CL ratio of 0.08 wt %), thus, lower catalytic activity towards the ε-CL polymerization is to be expected. Despite the slower kinetics and slightly lower PCL molar masses obtained, the original MMTMODL1 system showed significant eROP activity, clearly highlighting the great potential of this catalyst. Thus, subsequent studies on the influence of immobilization medium and biocatalyst drying and recovery were carried out with CALB immobilized on MMTMOD.

Recently, Sun et al. [12] emphasized the lack of systematic approaches in the current literature to the immobilization of CALB by adsorption in organic medium. Xin et al. [41] suggested that, as lipases are insoluble in the solvent, they can be only present within a thin aqueous film layer surrounding the support and, as such, higher immobilization efficiencies can be achieved by adsorption in organic media. It was concluded that the highest activity recoveries for CALB can be obtained from immobilizations conducted in more hydrophobic solvents [12].

Based on this concept, two different media and two different drying procedures were employed to test their respective effects on the resulting immobilized enzyme activity. CALB immobilization onto MMTMOD was performed both in phosphate buffer, as the aqueous medium, and toluene, as the organic medium, with a loading ratio of 7.5 mg protein per g of clay mineral. In each case, the resulting paste composed of enzyme adsorbed onto clay mineral was separated into two equal parts and dried by two different techniques, namely lyophilization and vacuum drying at 30 °C.

Hydrolytic activities of free and lyophilized CALB, as well as CALB-immobilized MMTMOD, were determined and are reported in Table 4. These catalytic systems (lipase/clay derivatives) were also tested for the eROP of ε-CL under typical conditions (10 mL of toluene, 3 mL of ε-CL and 300 mg CALB/clay derivative). Due to technical limitations for the evaporation of organic solvents in the lyophilizer, it was only possible to obtain a vacuum-dried catalyst from the lipase immobilization performed in toluene. No significant difference in final hydrolytic activities of the enzyme was observed, neither for the usage of two different media nor for the distinct drying procedures.

While the difference in terms of hydrolytic activity was not very important between MMTMODL-Aq-lyoph and MMTMODL-Org-dried catalysts, their performance for the synthesis of PCL chains were very different from each other. Although similar monomer conversion percentages (95% and 86%, respectively) were achieved, the MMTMODL-Org dried catalyst tended to produce much smaller PCL chains (3000 g/mol compared to 8000 g/mol for MMTMODL-Aq-lyoph). It can be concluded that while CALB immobilized onto MMTMOD in toluene led to an active catalyst system it exhibited lower performances compared to the immobilized lipase obtained in aqueous solution.

To explain such behavior, it is suggested that differences in water content play a key role. Indeed, the availability of water that is required to maintain the activity of the enzyme varies depending on the water partitioning among all the three components in the system: the organic medium, the enzyme, and the solid support [14,42]. In our case, when lipase was immobilized in organic media, it had a very limited amount of water as a thin film surrounding the enzyme available for its catalytic activity. When it was assayed for the hydrolysis of *p*NPB in buffer solution, it could find the water molecules necessary for its configuration change so that its active site was more accessible to the substrate. In this way it could reach similar hydrolytic activities to MMTMODL-Aq systems. On the other hand, during the catalyzed polymerization carried out in toluene, some motion restrictions may have occurred on account of the low hydration of the enzyme, causing a limited availability of its active site.

Additionally, the lyophilized catalytic system (MMTMODL-Aq lyoph) presented a lower content of “free” unbound water (ca. 1.7 wt %) than the system recovered by simple vacuum drying at 30 °C (MMTMODL-Aq dried), which contained more “free” water molecules (ca. 3.4 wt %) due to the difficulty to remove them from the clay mineral structure. As a consequence, as these water molecules could be involved as co-initiators in the polymerization, this could explain the lower molar masses of the obtained PCL chains (MMTMODL-Aq dried PCL, 3500 g/mol) (see Table 4).

It was then interesting to compare the performances of the clay-immobilized lipase to the NOV-435 commercial catalytic system. Firstly, it is worth pointing out that for reactions performed in similar conditions, the effective amount of enzyme varied. Indeed, 300 mg of MMTMODL-Aq-lyoph/dried or MMTMODL-Org dried catalysts contained approximately 2.5 mg of CALB protein (CALB/ε-CL ratio between 0.07 and 0.08 wt %), while 50 mg of NOV-435 introduced 5 mg of enzyme, corresponding to a CALB/ε-CL ratio of 0.17 wt %. It can be seen that our system, with a lower CALB/ε-CL ratio, was able to synthesize PCL chains with a molar mass of 8000 g/mol within 48 h, whereas NOV-435 containing twice the amount of enzyme, produced a PCL with a *M*_n_ value of 11,000 g/mol.

These results confirm the great potential of CALB immobilized on montmorillonite for ring opening polymerizations of lactones and clearly demonstrate that enzyme immobilization performed in aqueous medium followed by lyophilization lead to biocatalyst with highest eROP activity.

## 4. Conclusions

CALB immobilization on neat and organo-modified sepiolite and montmorillonite was performed. The influence of different parameters, e.g., enzyme loading, cross-linking with glutaraldehyde, solvent and drying process, on immobilization efficiency was investigated. The resulting CALB/clay catalysts were tested for both their hydrolytic activity and esterification potential for ε-caprolactone eROP. The optimal lipase content for immobilization was found to be 7.5 mg of protein per gram of clay while excessive enzyme loading results in lower immobilization efficiency. The characterization of the CALB/clay catalysts showed that, in all cases, lipase adsorption is limited to the external surface of clay minerals. The activity results confirmed that lipases immobilized on montmorillonite showed better performances compared to those immobilized on sepiolite and organo-modification of clays led to higher catalyst activity. In the case of MMTMOD, cross-linking by glutaraldehyde was found to enhance hydrolytic activity but had almost no influence on ε-caprolactone eROP activity. The eROP activity is higher for lipase immobilization performed in aqueous medium and PCL chains of higher molar mass are obtained when the biocatalyst is recovered and dried by lyophilization. With such optimal conditions, CALB/MMTMOD achieved 90% monomer conversion in 48 h, producing PCL chains with *M*_n_ of 10,000 g/mol. Interestingly, hydroxyl groups from the organo-modifier participate in the polymerization as co-initiators leading to PCL chain grafting and growth from the clay mineral surface, thus, allowing the preparation of clay/polyester inorganic/organic nanohybrids, as we recently reported [30,31].

## Figures and Tables

**Table 1:** Immobilization of lipase on different clay supports.

Catalyst	Loading (mg added protein/g clay)	Immobilization efficiency (%) ^1^	Hydrolytic activity (U/mg CALB) ^2^	Hydrolytic activity after GA treatment (U/mg CALB)
SEPL1a	7.5	88	37	13
SEPL2a	13.5	74	45	14
SEPL3a	27.0	56	35	16
SEPMODL1a	7.5	96	210	241
SEPMODL2a	13.5	98	199	222
SEPMODL3a	27.0	96	132	200
MMTL1a	7.5	99	117	132
MMTL2a	13.5	90	93	99
MMTL3a	27.0	73	91	80
MMTMODL1a	7.5	95	165	283
MMTMODL2a	13.5	97	145	246
MMTMODL3a	27.0	67	145	217
Free CALB	n/a	n/a	117	257

^1^ average standard deviation of mean immobilization efficiency is ±3%. ^2^ average standard deviation of mean hydrolytic activity is ±10%. n/a: not applicable.

**Table 2:** Influence of GA and lyophilization on the hydrolytic activity of CALB/organo-clays systems.

Catalyst	Loading (mg added protein/g clay)	Immobilization efficiency (%) ^2^	Hydrolytic activity before lyophilization (U/mg CALB) ^3^	Hydrolytic activity after lyophilization (U/mg CALB) ^3^
SEPMODL1b	7.5	98	210	60
SEPMODL1b G ^1^	7.5	98	287	117
MMTMODL1b	7.5	98	174	130
MMTMODL1b G ^1^	7.5	98	318	177

^1^ G was treated with glutaraldehyde. ^2^ average standard deviation of mean immobilization efficiency is ±3%. ^3^ average standard deviation of mean hydrolytic activity is ±10%.

**Table 3:** Catalytic performances of the immobilized systems for the ring-opening polymerizations (ROP) of ε-CL after 24 h of reaction.

Catalytic system	CALB/ε-CL ratio (wt %)	Monomer conversion (%) ^1^	*M*_n_ (g/mol) ^2^
NOV-435	0.17	93	16,000
NOV-435 in presence of MMTMOD	0.17	92	9,000
MMTMODL1b G	0.08	70	8,500
SEPMODL1b G	0.07	15	1,700

^1^ average standard deviation of monomer conversion values is ±5%. ^2^ determined by ^1^H NMR on an aliquot from the reaction medium.

**Table 4:** Effect of immobilization media and drying techniques on the hydrolytic activity and polymerization performance of CALB/MMTMOD (ε-CL conversion and polycaprolactone (PCL) molar masses).

Catalyst	Hydrolytic activity ^1^ (U/mg CALB)	Reaction time (h)	Monomer conversion ^2^ (%)	*M*_n_ (g/mol) ^3^
CALB	170	n/a	n/a	n/a
CALB LYOPH	127	n/a	n/a	n/a
NOV-435 in presence of MMTMOD	n/d	24	100	11,000
MMTMODL-Aq lyoph	126	48	95	8,000
MMTMODL-Aq dried	173	48	90	3,500
MMTMODL-Org dried	179	48	86	3,000

^1^ average standard deviation of mean hydrolytic activity is ±10%. ^2^ average standard deviation of monomer conversion values is ±5%. ^3^ determined by ^1^H NMR on the precipitated and dried final powder product. n/d: not determined; n/a: not applicable.

## Supplementary Materials

The following are available online at www.mdpi.com/2073-4360/8/12/416/s1, Figure S1: SEM images of SEPMOD after immobilization of CALB and treatment with GA at 8000× magnification; Figure S2: SEM images of MMTMOD after immobilization of CALB and treatment with GA at 8000× magnification.

## Abbreviations

The following abbreviations are used in this manuscript:

BSA	Bovine serum albumin
*p*NP	*p*-nitrophenol
*p*NPB	*p*-nitrophenyl butyrate
GA	glutaraldehyde
CALB	*Candida antarctica* lipase B
NOV-435	Novozym^®^-435, an acrylic resin-immobilized form of *Candida antarctica* lipase B
MMT	neat montmorillonite
MMTL	lipase (CALB) immobilized on neat montmorillonite
MMTMOD	organo-modified montmorillonite
MMTMODL	lipase (CALB) immobilized on organo-modified montmorillonite
MMTMODL-Aq	immobilization of CALB on organo-modified montmorillonite performed in aqueous medium
MMTMODL-Org	immobilization of CALB on organo-modified montmorillonite performed in organic solvent
SEP	neat sepiolite
SEPL	lipase (CALB) immobilized on neat sepiolite
SEPMOD	organo-modified sepiolite
SEPMODL	lipase (CALB) immobilized on organo-modified sepiolite
ε-CL	ε-caprolactone
PCL	poly(caprolactone)
ROP	ring opening polymerization
eROP	enzymatic ring opening polymerization
NMR	nuclear magnetic resonance
SEM	scanning electron microscopy
XRD	X-ray diffraction
