# Supplementary material for: Nanoclays for Lipase Immobilization: Biocatalyst Characterization and Activity in Polyester Synthesis
Source: Polymers (Basel). 2016 Dec 1;8(12):416. doi: 10.3390/polym8120416 (PMC6432194; doi:10.3390/polym8120416)
Supplement: Supplementary file 1 [file polymers-08-00416-s001.pdf]

Supplementary Materials: Nanoclays for Lipase Immobilization: Biocatalyst Characterization and Activity in Polyester Synthesis

Hale Öztürk, Eric Pollet, Vincent Phalip, Yüksel Güvenilir and Luc Avérous

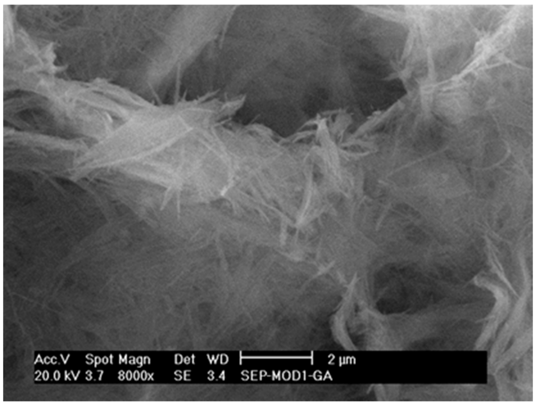

Figure S1. SEM images of SEPMOD after immobilization of CALB and treatment with GA, at 8000× magnification.

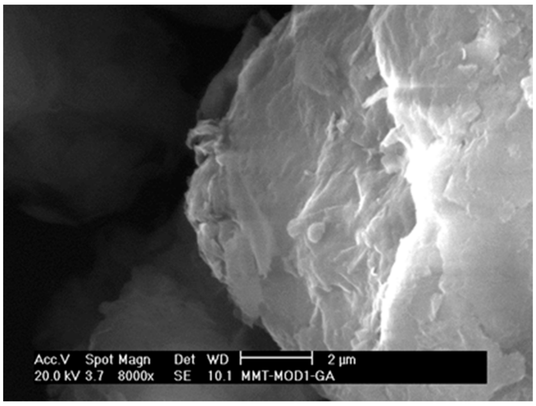

Figure S2. SEM images of MMTMOD after immobilization of CALB and treatment with GA, at 8000× magnification.
